# IL-12 and IL-18 Induction and Subsequent NKT Activation Effects of the Japanese Botanical Medicine Juzentaihoto

**DOI:** 10.3390/ijms9071142

**Published:** 2008-07-08

**Authors:** Kazuhiko Fujiki, Masanori Nakamura, Takako Matsuda, Mariko Isogai, Minako Ikeda, Yutaka Yamamoto, Mari Kitamura, Naoko Sazaki, Fumiatsu Yakushiji, Shinji Suzuki, Junji Tomiyama, Takashi Uchida, Ken Taniguchi

**Affiliations:** 1 Department of Internal Medicine, Tokyo Metropolitan Bokutoh Hospital, Tokyo, Japan; 2 Department of Oral Anatomy, Showa University, Tokyo, Japan; 3 Department of Cell Biology, Hiroshima University, Hiroshima, Japan; 4 Division of Rheumatic Diseases, Tokyo Metropolitan Bokutoh Hospital, Tokyo, Japan

**Keywords:** Japanese botanical medicine, Juzentaihoto, NKT, IL-12, IL-18

## Abstract

In this study, we first measured some cytokine concentrations in the serum of patients treated with Juzentaihoto (JTT). Of the cytokines measured interleukin (IL) -18 was the most prominently up-regulated cytokine in the serum of patients under long term JTT administration. We next evaluated the effects of JTT in mice, focusing especially on natural killer T (NKT) cell induction. Mice fed JTT were compared to control group ones. After sacrifice, the liver was fixed, embedded and stained. Transmission electron microscope (TEM) observations were performed. Although the mice receiving the herbal medicine had same appearance, their livers were infiltrated with massive mononuclear cells, some of which were aggregated to form clusters. Immunohistochemical staining revealed that there was abundant cytokine expression of IL-12 and IL-18 in the liver of JTT treated mice. To clarify what the key molecules that induce immunological restoration with JTT might be, we next examined *in vitro* lymphocyte cultures. Mononuclear cells isolated and prepared from healthy volunteers were cultured with and without JTT. Within 24 hours, JTT induced the IL-12 and IL-18 production and later (72 hours) induction of interferon (IFN)-gamma. Oral administration of JTT may induce the expression of IL-12 in the early stage, and IL-18 in the chronic stage, followed by NKT induction. Their activation, following immunological restoration could contribute to anti-tumor effects.

## 1. Introduction

Before the modern use of synthetic drugs, many medicines worldwide were derived from natural plant sources, including the nuts, roots and leaves. In Asian countries, especially in ancient China, many botanical materials had been adapted into medicines and these compositions have been described in old medicical encyclopedias.

Kampo originally means “Chinese style”, not only in medicine, but also for other Japanese words describing life styles. Kampo these days has come to mean “ethnical traditional medicine”. The principles of Kampo drugs were originally based on old Chinese traditional medicines and comprise about one hundred and fifty kinds of compositions of natural herbs and other natural products. Kampo medicine is generally characterized by its low frequency of severe adverse effects and is used in current clinical practice all over Japan and other countries.

Kampo products, for example, herbal extract granules for ethical use, were first included under the coverage of the Japanese public health insurance system in 1976 and currently, many clinical doctors in Japan apply these Kampo medicines along with Western chemically synthesized drugs [1].

Kampo traditional medicine is based on the long experience with its use in China and other oriental countries. The main theory of this medicine is based on the concept that diseases are caused from a disharmony of bodily flow and the aim of therapy is treating the patient as a body, not the disease. Consequently there can many ways to treat individual patients with the same disease.

“Sho”, the term of traditional Kampo medicine, refers to a particular pathological status of a patient evaluated by Kampo diagnosis, and is patterned according to the patient'sconstitution and symptoms, among other considerations [1]. Kampo preparations, including Japanese traditional botanical medicines, should be used after confirmation that it is suitable for the identified Sho of the patient.

Based on the particular diagnosis for Sho, some herbs and other natural materials should be compound into drugs. Originally they were taken as soluble extract in hot water just like tea, but now it can easily be taken as granular essence style drugs controlled under the Japanese Pharmacopoeia. Over ten pharmaceutical companies in Japan produce these granular style Kampo drugs for increased patient convenience [1].

Recently the use of alternative and complementary medicines in the Western countries has become more popular and some Kampo medicines can now be used in these countries. The use of Kampo style medicines is evolving how comprehensive medicine is practiced with established Western medical drugs.

Juzentaihoto (JTT), one of the Kampo Japanese herbal medicines was first described as SiQuan-Da-Bu-Tang in Daipinghuimin-Hejijufang (the Drug text book in Song Dynasty, 1151 A.D. in ancient China) and introduced into Japan in the Kamakura dynasty, about eight hundred years ago. The name of this drug originates from its Han–letters. This is composed with four parts of characters in the name, “Juzen” means perfect, “tai” means great, “ho” means supplement, and “to” means water drug, respectively, and it consists from ten crude components obtained from natural herbs (shown in Table 1 from the 2006 Japanese Pharmacopoeia) [2].

It has been mainly administrated to patients depressed or weakened by long illness to improve their general condition. The intended effect of this drug is to treat weakened conditions after illness, pale complexion, general fatigue, loss of appetite, night sweats, cold extremities, dry skin, dry mouth and anemia. Recently there have been some reports that JTT has anti-tumor effects and may prolong the survival of cancer patients [3–6]. Other reports describe that decreased occurrence of secondary carcinogenesis [7–11].

These pharmacological mechanisms by which JTT exerts its effects have not yet been clarified. One study reports JTT cause the induction of interleukin (IL)-12 and subsequent activation of natural killer T (NKT) lymphoid cells [12–13]. Other work mentions IL-18 induction as a possible result of JTT.

NKT cells are a type of lymphocyte characterized by their morphology which displays large granules in the plasma and has T cell receptors (TCR) with NK receptors, such as NK1.1. These cells have a role in both innate and adaptive immune responses [14]. Cytokines, such as IL-12 and IL-18, are important activators of NKT cells [15–18].

In this paper we describe experiments in designed to determine the ability of JTT to induce IL-12 and IL-18 and subsequent activation of NKT cells.

## 2. Materials and Methods

### 2.1. JTT

The herbal medicine, Juzentaihoto (JTT) was obtained from Tsumura Co. Ltd. (Tokyo, Japan) for this study. The quality of ten crude drugs shown in Table 1 is described by the 2006 Japanese Pharmacopeia. The patients who were treated in our hospital were taking JTT at a dosage of 7.5 g/day, 125 mg /kg/day or 4600 mg/m^2^ of. Mice were treated with 0.2% or 1.0% (w/w) drug mixed with chow. Assuming the mice weighed 25 g and ate 5 g per day, their exposures ranged from 1,200 mg/m^2^ to 6,000 mg/m^2^. The estimated dose levels in mice were calculated to bracket the estimated human exposure.

### 2.2. Patients

Patients have been treated in our hospital for many kinds of diseases, especially for liver function disorders. Twelve patients of liver cirrhosis with hepatocellular carcinoma were treated with/without JTT and compared to the same numbers of healthy volunteer and other disease (autoimmune diseases like Sjogren'ssyndrome) patients. All patients made informed-consent for our study following the Declaration of Helsinki, Ethical Principles for Medical Research Involving Human Subjects.

### 2.3. ELISA

To estimate the serum concentration of some cytokines, we used enzyme linked immunosorbent assay (ELISA) and followed the methods provided by the manufacturer. The source and sensitivity (lower limit of detection) of the ELISA kits are showed in Table 2.

### 2.4. Cell culture

Mononuclear cells were prepared from healthy volunteers of this hospital by lymph-prep^®^ (Immuno-Biological Lab., Takasaki, Japan) by recommended methods and dissolved in Dulbecco minimum essential medium (DMEM) with 5% fetal bovine serum (FBS) and some antibiotics. They were cultured with /without JTT and lymphocyte stimulation factor, like PMA (final concentration at 10ng/mL) for 24, 48, and 72 hours.

### 2.5. Mice

A total of 60 six-week old female specific pathogen free (SPF) C57BL/6 mice were purchased from Japan CLEA (Saitama, Japan) and housed and maintained at 24 °C and constant 60% humidity in the Animal Laboratory of Showa University. They were randomly divided into some groups, five mice per group, and examined after one-week standardizing diet prior to dosing. Mice were observed for changes in their body weight and appearance throughout study. Every group of mice were arranged volume of drugs on the schedule and sacrificed according to the experimental protection regulations of Showa University.

### 2.6. Pathological inspection

Mice were killed one, three, six and twelve months after administration. The body and liver were recorded at each examination. Formalin fixed and paraffin embedded liver tissue sections observed by hematoxylin eosin (HE) staining by optical microscope. Three pathologists independently done this observation and assessment of the drug effects and the representative were shown in the figures.

### 2.7. TEM

Transmission electron microscopic observation (TEM) has been done on the glutaraldehyde and hydrophil-resin embedded specimens by common methods.

### 2.8. Immunohistochemical staining (IHC)

After 4% (w/w) paraformaldehyde/PBS fixation, specimens were immersed in 5, 15, 30% sucrose/PBS and frozen in OCT compound. Cryosections were carried out and stained with primary antibody shown in Table 3. After washing, sections were incubated with the secondary antibodies at titer of 50%, biotinylated anti rat IgG made in goat (Vector Laboratories, Inc., USA, CA) and colored with ABC kit (Vector Laboratories, Inc., USA, CA) and DAB solution. Counter staining produced with methylgreen.

### 2.9. In situ hybridization mRNA analysis (ISH)

ISH was performed by use of a manual capillary actions system (MicroProbeTM® Staining System, Fisher Scientific, GA, U.S.A.). The sequence of the anti-sense oligo-nucleotide probe used for ISH were with the motifs as TCRVα14; (5′ AGG CTG AAC CTC TAT CCC CCA CCA CAC AGA 3′)products of Gene Reserch Lab® (Sendai, Japan).

### 2.10. Statistical study

Statistical tests were done with Student's t-test for multiple comparisons adjusted for in the analysis.

## 3. Results

### 3.1. Serum cytokines

**Figure 1.**
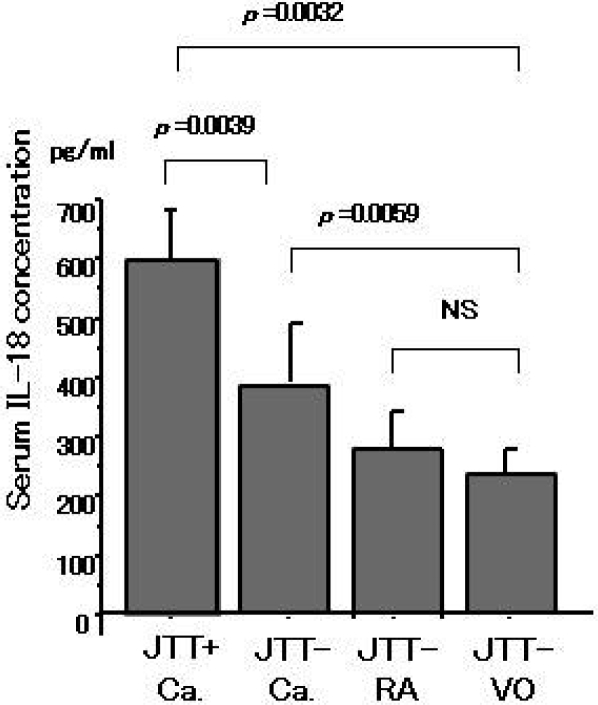
Serum concentrations of IL-18 from liver cancer (Ca), autoimmune disease (RA) patients and healthy volunteers (VO). JTT administration (JTT+) may result in induction of IL-18.

Serum from patients treated with JTT medicine showed no changes in the concentration of several serum cytokines (IL-1, IL-2, IL-4, IL-6, IL-12, TNF-α, IFN-γ, data not shown). These cytokines were undetectable by the ELISA in this study. These results of serum IL-18 are shown in Figure 1. IL-18 levels were increased in patients treated with JTT for over one year, compared with sera from non-treated patients and healthy volunteers. There is no relation between IL-18 and CRP which one of the clinical indicators of inflammatory diseases (data not shown). For comparison purposes, the serum levels of IL-18 from patients with autoimmune diseases (not treated with JTT) are included in Figure 1.

**Figure 2.**
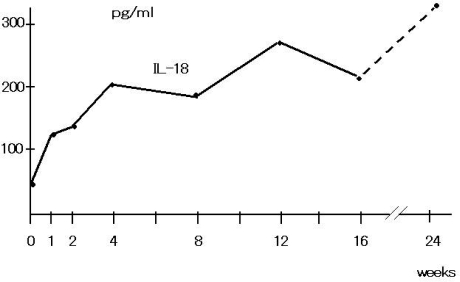
Serum Cytokine from the JTT Administrated Patient, IL-18 increased, other cytokines (IL-1, IL-2, IL-4, IL-6, IL-12, TNF-a, IFN-g, Data not shown) were not detectable in patient'sserum.

A representative time course (0–24 weeks) of the changes in serum IL-18 concentration from JTT treated patients is shown in Figure 2. IL-12 concentration was also monitored along with IL-18, but there was no remarkable changes in IL-12 levels.

### 3.2. Cell culture

**Figure 3.**
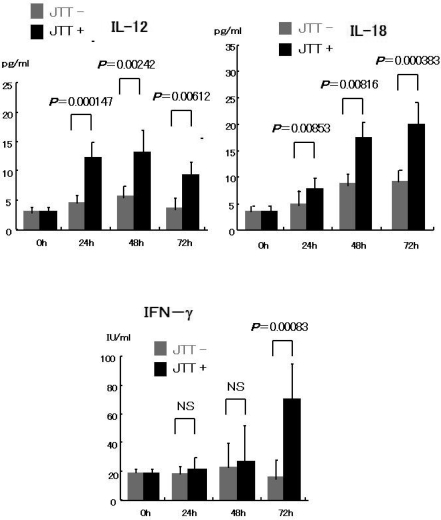
Cytokine from Cultured PBMC with/without JTT. PBMC cultured with/without JTT in DMEM with 5% Bovine Calf Serum. JTT induced IL-12 and IL-18 production following IFN-g within 72 hours *in vitro*.

The peripheral mono nuclear cells prepared from healthy volunteers were cultured with JTT. It resulted in the high concentration of IL-12 and IL-18 production in the supernatant. Induction of cytokines occurred within 24 hours in this culture, and continued at least to 72 hours. IFN-γ production increased following after IL-12 and IL-18 induction. These results are shown in Figure 3.

### 3.3. Pathological changes in liver

**Figure 4.**
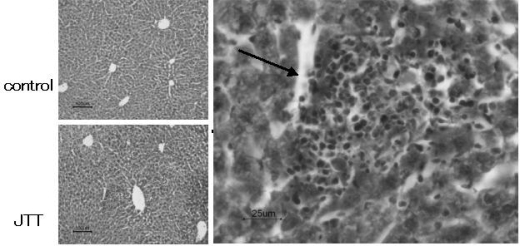
Mononuclear cell infiltration with JTT treatment. Liver was infiltrated by massive mononuclear cells in JTT treated mice and some of which were aggregated to clusters (arrow head).

Although there are no specific changes in appearance, body weight and liver weight of JTT treated mice (data not shown), these livers were infiltrated by massive mononuclear cells, some of which were aggregated to form clusters (Figure 4).

### 3.4. TEM findings

**Figure 5.**
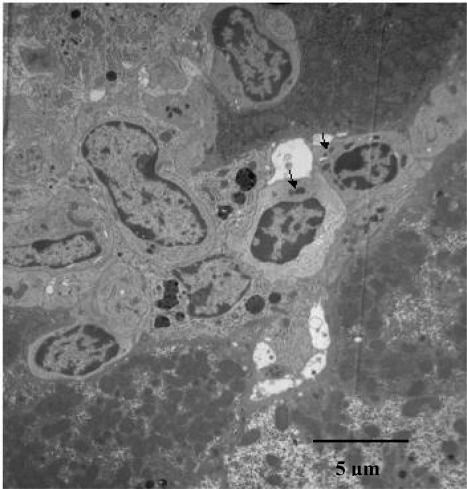
Aggregated lymphocyte with JTT. There are some large granular lymphocytes, some of which have electron high dense granules in the cytoplasma, in the liver of JTT treated mice (TEM scan methods).

TEM observation indicated that there are some large granular lymphocytes, some of which have electron high dense granules in their cytoplasm, in the liver of JTT treated mice (Figure 5). The findings of these mononuclear cells are consistent with them being NKT cells.

### 3.5. NKT infiltration in the JTT treated mice Liver

**Figure 6.**
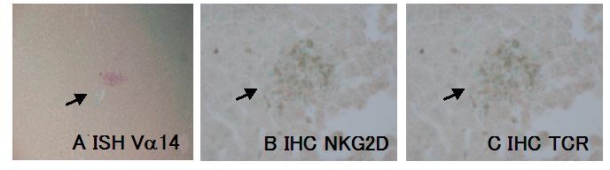
TCR Va14 expression on the surface of infiltrated mononuclear cells, detected by ISH (A) and NKG2D (B), TCR (C) by IHC method.

The IHC results showed that many of the infiltrating mononuclear cells abundantly expressed NKG2D, a reported selective NK marker and T cell receptor (TCR) on their surface (shown in Figure 6).

### 3.6. ISH for Va14

mRNA in situ hybridization method for Vα14, the specific T cell receptor of NKT, indicated that there were many Vα14 positive NKT cells infiltrating into the liver of JTT treated mice and forming the cluster.

### 3.7. IL-12 expression on the JTT treated mice liver

**Figure 7.**
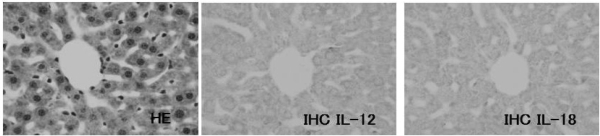
Expression of cytokines. The liver tissue from the control mice. They have expressed little of cytokines.

**Figure 8.**
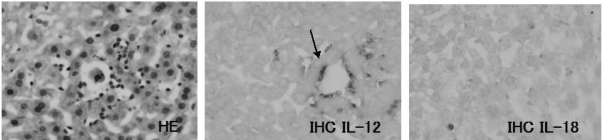
Expression of cytokines. The lice tissue from the 1% JTT short term treated mice. There are no expression of cytokines on some cells (arrow head).

**Figure 9.**
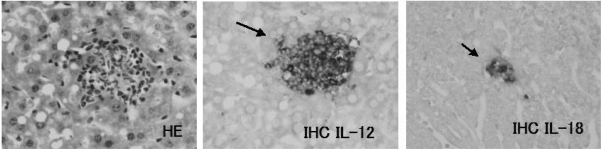
Expression of cytokines of the liver tissue from the 0.2% JTT long term treated mice. There are expression of cytokines on some cells which aggregated into clusters (arrow head).

**Figure 10.**
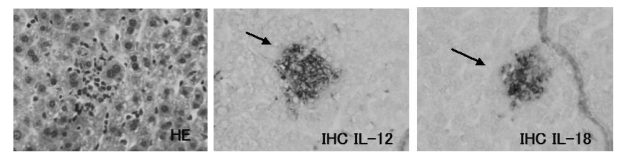
Expression of cytokines of the liver tissue from the 1% JTT long term treated mice. There are abundant expression of cytokines on some cells which aggregated into clusters (arrow head).

IHC analysis for the cytokine IL-12 revealed abundant expression of IL-12 on the cells infiltrated into liver of JTT treated mice. The expressions of IL -12 increased with longer drug exposure time (Figures 7–10)

### 3.8. IL-18 expression on the JTT treated mice liver

**Figure 11.**
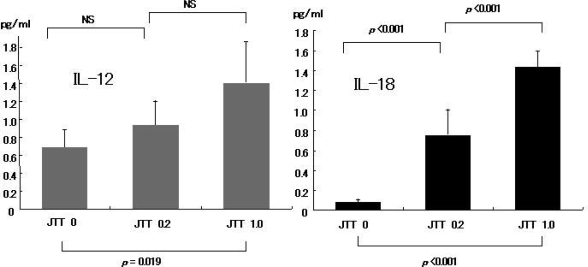
Serum Cytokine from Long Term JTT treated Mice. JTT long term treatment included up-regulation of serum cytokine concentration of IL-12, and IL-18. These results indicate that significant increase related with JTT concentration.

IHC analysis for IL-18 expression revealed abundant cytokine levels was present on the cells from JTT treated mice liver. IL-18 expression had the same appearance with IL-12 (Figures 7–10). Long term JTT treatment induced up-regulation of serum cytokine concentration of IL-12, and IL-18. These results indicate that significant increase related with JTT concentration. (Figure 11)

## 4. Discussion

Herbal prescriptions like Japanese Kampo medicine have been recognized for their potential and clinical usefulness. Recently, Juzentaihoto (JTT), one of these Japanese botanical medicines, has been reported to experimentally display anti-tumor effects against liver metastasis. Through this study we attempted to reveal a possible pharmacological mechanism of JTT's anti-tumor effects.

First we compared the serum cytokines from treated and non-treated patients. JTT treated patients in our study appeared to have high serum concentrations of IL-18, compared with non-treated patients, but the levels of IL -18 showed no relation to their disease status, as indicated by serum CRP. IL-18 was also up-regulated in serum from non JTT treated patients with liver cirrhosis, but to a lesser extent than in subjects treated with JTT. Although these patients do not formally have hepatocellular carcinoma, they represent a patient population at risk for developing liver cancer and may be viewed as pre-cancerous, which might be the reason for the elevated IL-18 levels in the untreated cirrhotic patients. IL-18 from the serum of autoimmune disease patients were also slightly up-regulated compared with healthy volunteers. This might be from activated immuno-reaction, but there is still question why the other cytokines were not changed. The evaluation of cytokines was performed with commercial ELISA kits according to manufacturer's methods. These kits have satisfactory potential of detection of cytokine in these sensitivity (most have pg/mL sensitivity) and specificity. We were unable to detect measureable levels of several cytokines in this study, but since many cytokines are transiently expressed *in vivo*, and decline rapidly following induction, the fact that we could not detect several of the cytokines tested is not surprising.

As the evaluation results of serum cytokines were not detected sufficiently in patient sera, we next constructed two experimental models. One is the cell culture method for *in vitro* cytokine production; the other is an animal model to look for tissue expression of cytokines *in vivo*.

Human lymphocyte cultures incubated with JTT produced elevated levels of both IL-12 and IL-18. One possible additional source of increased cytokines (i.e., IL -18) in the peripheral blood could be from mononuclear cells in tissues. INF-γ production was also up-regulated following after IL-12 and IL-18. Up-regulation of this pathway could be involved in the anti-tumor effects of JTT administration via TH1 lymphoid effects.

Treatment of mice with JTT produced no significant changes for growth and liver function, yet in this study there were some interesting pathological results in the livers of JTT treated mice. Massive mononuclear cell infiltration and some of these were aggregated in clusters. These aggregated mononuclear cells are mainly considered NKT cells for their specific surface markers and for their characteristic intracellular structures.

This report also shows that JTT administration might induce IL-12, which has been originally described as a NKT cell stimulatory factor, as well as IL-18 expression. Our results revealed many mononuclear cells, including dendritic cells and Kupffer cells in liver tissue of JTT treated mice and expressed abundant IL-12 and 1L-18.

These cytokine expressions induced by JTT might be the reason of massive mononuclear cells, especially NKT cells infiltration and their activation, following immunological restoration could contribute to anti-tumor effects.

## Conclusions

Although there is some vagueness concerning the ingredients and pharmacological efficiency of the ten crude components of the Japanese botanical medicine Juzentaihoto, the consumption of natural herbs has certain inductive effect on NKT cells. Further studies for elucidate the efficacy of natural herbs for disease treatment are recommended. Cancer patients are always at risk of developing a secondary malignancy, and JTT may help to decrease this risk without severe adverse effects. JTT, one of Japanese herbal medicine administration might be the alternative strategy of treatment for cancer patients to prevent secondary oncogenesis.

## Figures and Tables

**Table 1.** Botanical origins of ten crude drugs of JTT from the 2006 Japanese Pharmacopoeia.

Crude Drugs	Scientific Name of Botanical Origin	Ratio
*Astragali Radix*	*Astragalus membranaceus Bunge* or *A. mongholicus Bunge*	3.0
*Cinnamomi Cortex*	*Chinnamomum cassia Mlume* or other species of the same genus	3.0
*Rehmanniae Radix*	*Rehamannia glutinosa Liboschitz var.*	3.0
	*Purpurea Makino* or *A. glutinosa Liboschitz*	
*Paeoniae Radix*	*Paeonia lactiflora Pallas* or allied plants	3.0
*Cnidii Rhizoma*	*Cnidium officinale Makino*	3.0
*Atractylodis*	*Atractylodes launcea De Candolle or A. chinesis Koidzumi*	3.0
*Lanceae Rhizoma*		
*Angelicae Radix*	*Angelica acutiloba Kitagawa* or allied plants	3.0
*Ginseng Radix*	*Panax ginseng C.A. Meyer*	3.0
*Hoelen*	The sclerotium of *Poria cocos Wolf*	3.0
*Glycyrrhizae Radix*	*Glycyrrhiza uralensis Fischier, G. grabra Linne*, or other species of the same genus	

**Table 2.** Commercial ELISA kits used in this study (h: human, m: mice).

Cytokine	Kit code	Production	**Sensitivity**
hIL-1	RPN5971	GE Imagination at Work	>4 pg/mL
hIL-2	JIMRO IL-2	Otsuka Pharma. Tokyo, Japan	>50 pg/mL
hIL-4	S-4050	R & D Systems Inc. MN, U.S.A.	>15 pg/mL
hIL-6	D-6050	R & D Systems Inc. MN, U.S.A.	>8 pg/mL
hIL-12 (supernatant)	EHIL12	Pierce Biotech. Inc. IL, U.S.A	>3 pg/mL
hIL-12 (serum)	P1200	R & D Systems Inc. MN, U.S.A.	>8 pg/mL
hIL-18	#7260	Med. Biologic. Lab. Nagoya, Japan	>12.5 pg/mL
hTNF-α	QTA00B	R & D Systems Inc. MN, U.S.A.	>0.5 pg/mL
hIFN-γ	IM1743	Immunotech. Co. Marseille, France	>0.4 IU/mL
mIL-12	M1270	R & D Systems Inc. MN, U.S.A.	>2.5 pg/mL
mIL-18	#7625	Med. Biologic. Lab. Nagoya, Japan	>25 pg/mL

**Table 3.** Primary antibodies used in this study

Antigen	Clone	Production
TCRαβ	MO073207	Becton Dickinson Co. (NJ, U.S.A.)
NKG2D	191004	TECHNE (Staffordshire, U.K.)
IL-12	C15.6	Biosource International Inc. (CA. U.S.A.)
IL-18	SC9464	Santa Cruz Biotech. Inc. (CA. U.S.A.)
